# Achievements of the Australian Access to Allied Psychological Services (ATAPS) program: summarising (almost) a decade of key evaluation data

**DOI:** 10.1186/s13033-016-0092-4

**Published:** 2016-09-26

**Authors:** Bridget Bassilios, Angela Nicholas, Lennart Reifels, Kylie King, Justine Fletcher, Anna Machlin, Maria Ftanou, Grant Blashki, Philip Burgess, Jane Pirkis

**Affiliations:** 1 Centre for Mental Health, Melbourne School of Population and Global Health, The University of Melbourne, Victoria, 3010 Australia; 2 School of Public Health, The University of Queensland, Herston, QLD 4006 Australia; 3 Nossal Institute for Global Health, Melbourne School of Population and Global Health, University of Melbourne, Victoria, 3010 Australia

**Keywords:** Access to Allied Psychological Services, Mental health service, Primary health care, Mental health policy

## Abstract

**Background:**

Introduced in July 2001, Australian Access to Allied Psychological Services (ATAPS) was the inaugural national policy initiative to provide community access to government-funded psychological services in primary care. Our aim was to examine the achievements of ATAPS in relation to its stated objectives using a set of indicators that largely drew on data from a minimum data set that we designed for the evaluation of ATAPS.

**Methods:**

We used de-identified professional-, consumer- and session-level data from the minimum dataset, and secondary analyses of our quantitative and qualitative data collected for a series of specific evaluation studies. Available data covered the period from 1 July 2003 to 31 December 2012.

**Results:**

Approximately 350,000 referrals were made to the ATAPS program over the 9.5 year analysis period, 79 % of which resulted in services. Over 1.4 million sessions were offered. Overall, 29 % of consumers were male, 4 % children, and 3 % Aboriginal people; 54 % of consumers had depression and 41 % an anxiety disorder; at least 60 % were on low incomes; and around 50 % resided outside of major cities. The most common interventions delivered were cognitive and behavioural therapies. Selected outcome measures indicated improvement in mental health symptoms.

**Conclusions:**

Access to Allied Psychological Services achieved its objectives within a decade of operation. The program delivered evidence-based services to a substantial number of consumers who were disadvantaged and historically would not have accessed services. Importantly, where data were available, there were indications that ATAPS achieved positive clinical outcomes for consumers. This suggests that ATAPS carved an important niche by successfully addressing unmet need of hard-to-reach consumers and through means that were not available via other programs. It will be interesting to see the effects from July 2016 of the reform of ATAPS, which will see ATAPS subsumed under psychological services commissioned by regional primary care organisations.

## Background

Australia’s mental health system is complex but can usefully be described according to four categories of services: universal mental health promotion and mental illness and suicide prevention services, primary care and/or general health services, specialised clinical services (e.g., bed-based services, psychiatric emergency care and community services/teams) and mental health community support sector services (e.g., family and carer services; personalised support services; group support services; mutual support and self-help; education, employment and training; care co-ordination) [1]. There is good international evidence for the treatment of common mental disorders among adults in the primary care setting [2–4]. Since 2001 the Australian Government has been funding primary mental health care in a targeted manner through its Access to Allied Psychological Services (ATAPS) program. Since 2006, ATAPS has been complemented by population-based primary mental health care subsidised via the Medicare Benefits Schedule, Australia’s publicly funded universal health care scheme [5]. Prior to ATAPS, access to primary mental health services was limited to consumers who were able to pay a fee-for-service and/or those who received a partial rebate through a private health insurance provider.

Access to Allied Psychological Services was introduced in a series of funding rounds over 2 years from July 2001. ATAPS was the inaugural national policy initiative to provide community access to government-funded psychological services in primary care. ATAPS enabled people with high prevalence disorders to consult with mental health professionals (psychologists, social workers, mental health nurses, occupational therapists and Aboriginal and Torres Strait Islander health workers with specific mental health qualifications) for low-cost or free evidence-based mental health care, usually on the basis that they were referred by a GP. This care was delivered in up to 12 (or 18 in exceptional circumstances) individual sessions [6]. Consumers were also able to access a further 12 sessions in a group format [7]. Review by the referring GP was essential after each block of six sessions and/or the final session.

Access to Allied Psychological Services was introduced to improve treatment rates following findings from the 1997 Australian National Survey of Mental Health and Wellbeing (NSMHWB) indicating that one in five Australians had a diagnosed mental disorder in the preceding 12 months, with anxiety and affective disorders being the most common classes of mental disorders [8]. The 1997 NSMHWB also found that around two-thirds of people with common mental disorders were not receiving treatment [9, 10]. Of those who did seek treatment, GPs were the most commonly consulted health professionals [11].

Access to Allied Psychological Services became a key part of primary mental health care delivery in Australia. The program took the form of a series of projects that were originally managed by a network of local primary mental health organisations known as Divisions of General Practice (‘Divisions’). From July 2011 to July 2012, the Australian Government funded the transition from Divisions into 61 Medicare Locals nationwide with the view “to improve coordination and integration of primary health care in local communities, address service gaps, and make it easier for patients to navigate their local health care system” [12]. Although this paper presents data up to December 2012, developments beyond this date and looking forward are noteworthy. By July 2015, a review of Medicare Locals resulted in the announcement of a new structure for primary care organisations comprising 30 Primary Health Networks in an effort to improve efficiency and effectiveness of the health system and integration between health professionals [13]. Historically, Divisions and Medicare Locals received, and Primary Health Networks currently receive, capped Government funding to broker or provide local ATAPS mental health services. As a result of a major review of all Australian mental health services completed in 2014 [14], the mental health system is again undergoing major reform and from July 2016, Primary Health Networks will receive a flexible funding pool to commission primary care psychological treatment (subsuming ATAPS amongst other programs) within a stepped care approach according to local population mental health needs [15].

Over the life of ATAPS, it underwent various refinements in response to policy directives and community needs. The most significant of these occurred at about the time another primary mental health care initiative, the Better Access to Psychiatrists, Psychologists and GPs (Better Access) initiative, was introduced in 2006. For the consumer, ATAPS and Better Access appear reasonably similar; Better Access also enables consumers to consult with mental health professionals on referral from a GP. The difference is that funding for Better Access is delivered through the Medicare system [16], which means that unlike ATAPS it is not capped. As a result, since 2008, ATAPS evolved into a program that continued to provide the original ATAPS services (‘Tier 1 initiative’) to people with a diagnosed mental disorder, but became more specialised and provided tailored service delivery for particular hard-to-reach groups (‘Tier 2 initiatives’). Tier 2 initiatives specifically targeted women with perinatal depression; individuals who had attempted, or were at risk of, suicide and self-harm; people impacted by extreme climatic events; people experiencing, or at risk of, homelessness; people in rural and remote locations; Aboriginal and Torres Strait Islander people; and children who had, or were at risk of developing, a psychological disorder. Medicare Locals were granted additional flexible Tier 2 funding to enhance their capacity to address the needs of these at-risk groups and to provide increased service flexibility or innovation [17], as outlined in Table 1. In order to facilitate access to services for marginalised groups who may not have had a GP, the introduction of Tier 2 initiatives resulted in medical professionals other than GPs (psychiatrists and paediatricians) and non-medical professionals (e.g., ATAPS mental health professionals, school counsellors, school principals, and non-government organisations) being able to refer consumers to ATAPS. However, there was an expectation that consumers initially referred by non-medical professionals were linked to a GP who would develop and review their mental health care plan.

**Table 1:** Tier 2 ATAPS flexibilities (Adapted from Reifels et al. [17])

ATAPS Tier 2 initiative	Key flexibilities
Perinatal depression (‘Perinatal depression’) introduced April 2008	Provisional referral option (midwives, obstetricians, maternal and child health nurses) New intervention (family-based intervention)
Telephone-CBT Pilot (‘T-CBT’) July 2008–June 2010	New telephone modality (telephone-, web- and video conferencing-based modalities now available across all ATAPS initiatives)
Specialist suicide services (‘Suicide prevention’) piloted October 2008–June 2011; expanded to all medicare locals in July 2011	Provisional referral option (community mental health services, psychiatrists, emergency departments) No diagnosis requirement No limit on session numbers Shorter more intensive support model
Victorian Bushfires 2009 (‘Bushfire’) introduced February 2009	Provisional referral option (bushfire case managers) Originally no diagnosis requirement Originally no limit on session numbers
Aboriginal and Torres Strait Islander introduced July 2010	Provisional referral option (non-government organisations) New outreach modality New intervention (narrative therapy, available across all ATAPS initiatives)
Children with mental disorders (‘Child’) introduced July 2010	Provisional referral option (school counsellors, school principals, directors of early childhood services) New interventions (family-based intervention, parent training in behaviour management, play therapy) Flexible session mode: child alone, parent(s) alone, child and parents, child in group, parent(s) in group, child(ren) and parent(s) in group
People experiencing or at risk of homelessness (‘Homelessness’) introduced July 2010	Provisional referral option (non-government organisations) New outreach modality (including mobile clinics)
Rural and remote introduced July 2010	New outreach modality
Floods and cyclone Yasi in Queensland, New South Wales and Victoria 2010–2011 (‘Floods and cyclone Yasi’) introduced January 2011	Provisional referral option (Centrelink social workers, State Mental Health Services, client self-referral) Originally no diagnosis requirement Originally no limit on session numbers for people diagnosed with a disorder through a mental health treatment plan

*ATAPS* Access to Allied Psychological Services; *CBT* cognitive behavioural therapy

As a result of the above changes to the ATAPS program, its original broad objective of improving the quality of primary mental health care [18] was also refined in 2012. The refined objectives of the ATAPS program [7] were to:

- offer referral pathways for GPs to support their role in primary mental health care;
- promote a team approach to the management of mental disorders;
- target services to those individuals requiring primary mental health care who were not likely to be able to have their needs met through Medicare-subsidised mental health services;
- offer non-pharmacological approaches to the management of common mental disorders;
- produce better outcomes for individuals with common mental disorders through offering evidence-based short-term psychological interventions within a primary care setting; and
- complement other fee-for-service programs and address service gaps for people in particular geographical areas and population groups.

The authors have been involved in the evaluation of the ATAPS program since 2003. The evaluation model was unique in that it evolved alongside the ATAPS program. This enabled us to examine how the program progressed over time, and the impact it had on the Australian mental health care service delivery landscape. The evaluation design was multifaceted and responsive to changes in the program, incorporating a range of different data sources and approaches to analysis. The evaluation relied on routinely collected data from a national web-based minimum dataset, which provided information regarding uptake of the program, characteristics of consumers, types of psychological services delivered and consumer outcomes. We supplemented this quantitative data with qualitative data sources (e.g., program documentation; organisation-level evaluation reports; a forum with project staff; and topic-specific surveys of, and interviews with, various stakeholders), which provided depth of information. This paper is mainly based on quantitative data from a larger report [19] and supplemented by secondary analyses of our quantitative and qualitative data, which were used to evaluate the achievements of ATAPS over (almost) one decade in terms of its refined stated objectives.

## Method

We used provider-, consumer- and session-level data (entered or uploaded by Medicare Locals or providers) from the minimum dataset to develop proxy indicators of the achievement (or non-achievement) of each ATAPS objective. These indicators and their associated data sources in relation to each objective are summarised in Table 2.

**Table 2:** Indicators and data sources used to examine whether each of the ATAPS objectives has been achieved

Objectives	Indicators	Data sources
Offer referral pathways for GPs to support their role in primary mental health care	Participation by GPs (and other referring professionals) Number of referrals Number of sessions	Minimum dataset
Promote a team approach to the management of mental disorders	Participation by GPs (and other referring professionals) Participation by mental health professionals Provider perspectives of benefits of ATAPS	Minimum data set Secondary analysis of qualitative data
Target services to those individuals requiring primary mental health care who are not likely to be able to have their needs met through Medicare subsidised mental health services	Consumer characteristics Session characteristics Uptake of Tier 2 initiatives	Minimum dataset
Offer non-pharmacological approaches to the management of common mental disorders	Consumer diagnoses Interventions	Minimum dataset
Produce better outcomes for individuals with common mental disorders through offering evidence-based short-term psychological interventions within a primary care setting	Consumer outcomes Consumer diagnoses Interventions	Minimum dataset
Complement other fee-for-service programs and address service gaps for people in particular geographical areas and population groups	Program uptake Consumer characteristics Session characteristics (all compared to Better Access)	Minimum dataset Secondary analysis of Better Access data

*ATAPS* Access to Allied Psychological Services; *GP* general practitioner

The minimum dataset advanced over time in response to organisations’ stated needs, the introduction of new services and the transitions from Divisions to Medicare Locals and from Medicare Locals to Primary Health Networks. Data from the minimum dataset were available for the period from 1 July 2003 (when the minimum dataset was first ‘rolled out’) to 31 December 2012 and were extracted on 1 April 2013.

We also conducted secondary analyses of some of our earlier qualitative and quantitative data and our evaluation of Better Access.

The research questions in relation to each objective, details of corresponding indicators and the ways in which these were analysed are described below.

### Did ATAPS offer referral pathways for GPs to support their role in primary care?

The overall uptake of ATAPS was considered to provide evidence of the existence of referral pathways for GPs (and other referring professionals). The minimum dataset captured the number and types of professionals referring consumers to ATAPS, the number of mental health professionals delivering services, and de-identified consumer-level and session-level information. Frequency data on participation by GPs (and other referring professionals), the number of referrals made and sessions delivered were generated to evaluate this objective.

### Did ATAPS promote a team approach to the management of mental disorders?

We used participation by GPs and other referring, and treating mental health, professionals as indicators of whether ATAPS had promoted a team approach to the management of mental disorders. We also conducted a secondary analysis of some of our earlier qualitative data on the experiences of providers.

### Did ATAPS target services to those individuals requiring primary mental health care who were not likely to be able to have their needs met through Medicare-subsidised mental health services?

We interpreted individuals not likely to have their needs met through Medicare-subsidised mental health services as being groups of people who are typically disadvantaged, such as those residing in rural and remote or socio-economically disadvantaged locations, Indigenous people and children. Males who do not typically access (mental) health care, and have been identified as a hard-to-reach group in the National Male Health Policy [20], were also considered to be in this group. Therefore, we used consumer (e.g., age, gender, Indigenous status, postcode, low income status, previous psychiatric care) and session characteristics (e.g., consumer out-of-pocket fees), as well as the uptake of Tier 2 initiatives as indicators of whether ATAPS had targeted services to people who were not likely to have had their needs met through Medicare-subsidised mental health services, noting that the Tier 2 initiatives were in fact intentionally introduced to target disadvantaged groups. Note that the ATAPS data collection does not define low income according to an individual’s or family’s income amount; instead assignment of low income is based on the referrer’s judgement that takes into account comparative levels of income and evidence such as health care card or pension status.

Consumers’ postcodes at the time of referral were used to classify region of residence according to the Australian Standard Geographical Classification (ASGC) Remoteness Structure [21]. The ASGC Remoteness Structure is one of the seven structures that comprise the Australian Standard Geographic Classification (ASGC) produced by the Australian Bureau of Statistics (ABS) using census data. The ASGC classifies geographical areas into six categories (of which the sixth was not relevant for our purposes): (1) Major cities; (2) Inner regional; (3) Outer regional; (4) Remote; (5) Very remote; and (6) Migratory. ABS mapping files that provide the proportion of the Australian population within a given postcode allocated to a particular remoteness area were used to map remoteness areas for ATAPS consumers. Referrals made prior to 2009 were based on 2006 mapping and referrals from 2009 onwards on 2011 mapping.

### Did ATAPS offer non-pharmacological approaches to the management of common mental disorders?

We note that ATAPS providers by virtue of their professions (psychologists, social workers, mental health nurses, occupational therapists and Aboriginal and Torres Strait Islander health workers), were not qualified to prescribe psychotropic medication. Therefore, while this precise objective is rhetorical as a research question, for the purpose of our evaluation, we interpreted the objective more broadly as being about the types of interventions delivered via ATAPS in order to manage the high prevalence disorders of depression and anxiety. According to the Operational Guidelines [7], providers were expected to deliver evidence-based interventions such as cognitive behavioural therapy, psycho-education, relaxation, skills training, interpersonal therapy and narrative therapy. Therefore, frequency data on the nature of interventions were extracted from the minimum dataset and were used to determine whether these interventions were delivered, rather than as indicators of whether ATAPS had met the objective as stated. We also extracted consumer diagnostic frequency data to confirm that people with common mental disorders were accessing ATAPS. These data were supplemented with frequency data on the use of medication (prescribed by referring medical professionals) by ATAPS consumers, which were also extracted from the minimum dataset.

### Did ATAPS produce better outcomes for individuals with common mental disorders through offering evidence-based short-term psychological interventions within a primary care setting?

We interpreted better outcomes for individuals as improvements in their mental health symptoms as assessed using standardised outcome measures. Therefore, consumer outcome data available in the minimum dataset were used to address this question. It was beyond the scope of this paper to report on the entire suite of outcome measures used. Therefore, we selected the two most commonly used measures, namely the Kessler-10 (K-10) [22] and the Depression Anxiety and Stress Scales (DASS) [23], and two Tier 2 initiative-specific measures: the Modified Scale for Suicidal Ideation (MSSI) [24, 25] for the suicide prevention initiative and Edinburgh Postnatal Depression Scale (EPNDS) [26] for the perinatal depression initiative.

The K-10 [22] is a 10-item self-report measure of non-specific psychological distress, with a total score above 19 indicating one of three levels of psychological distress from mild (20–24) to severe (30–50). The DASS [23] comprise 7–14 items on each subscale, depending on whether the DASS-21 or -42 is used. Scores above 9, 7 and 14 on the depression, anxiety and stress subscales, respectively, indicate one of four levels of symptom severity from mild to extremely severe. The MSSI [24, 25] is an 18-item measure with a total score indicating one of three levels of suicidal ideation from low (0–8) to severe (21+). The EPNDS [26] includes 10 self-rated items to assess postnatal depression, with scores above 10 indicating the presence of depressive illness of varying severity. All of these measures have demonstrated sound psychometric properties [22, 25–28].

Consumer outcomes were analysed using paired t-tests to examine the difference between mean pre- and post-treatment scores on the K-10, DASS, EPNDS and MSSI, with positive differences indicating improvement. Consumers who did not have a ‘matched pair’ of pre- and post-treatment scores were excluded. Effect sizes were calculated by dividing the difference in means by the difference in standard deviations.

We also calculated mean session data in order to confirm the short-term nature of interventions.

### Did ATAPS complement other fee-for-service programs and address service gaps for people in particular geographical areas and population groups?

The complementarity of ATAPS was interpreted as its accessibility to groups of consumers who are unable to access the much larger Better Access program. Therefore, we compared certain features (e.g., uptake, consumer and service characteristics) of the two programs using secondary analyses of the evaluation of the latter and an earlier study which examined the reciprocal impact of the two programs [29]. The extent to which ATAPS addressed service gaps was beyond the scope of the current paper.

## Results

### Did ATAPS offer referral pathways for GPs to support their role in primary mental health care?

Data from the minimum dataset showed that ATAPS offered referral pathways for GPs, thereby strengthening their role in primary mental health care. Our best estimate is that around 32,000 providers made referrals to ATAPS between 1 July 2003 and 31 December 2012, although we acknowledge that this may be something of an over-count because of limitations in some Medicare Locals’ ability to uniquely identify providers across financial years and/or across sub-programs. Around 90 % of referring providers were GPs. A clear increase in the number of referring providers across financial years (from 1716 in 2003–2004 to 13,157 in 2011–2012) was observed as Tier 1 services became more commonplace and as Tier 2 sub-programs became available.

**Fig. 1:**
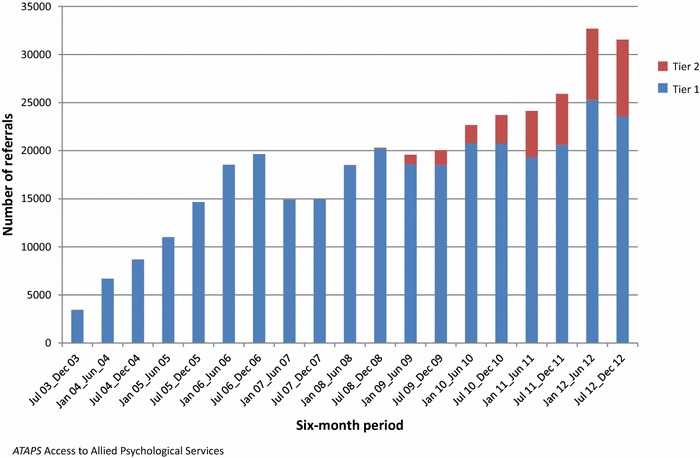
Number of referrals by ATAPS initiative in six-month blocks, July 2003–December 2012

**Fig. 2:**
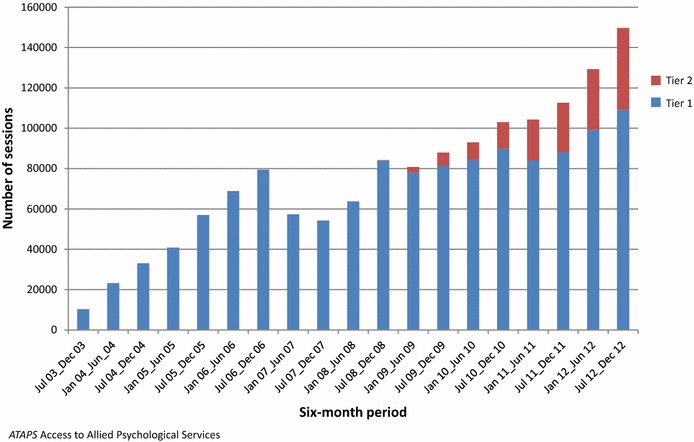
Number of sessions by ATAPS initiative in six-month blocks, July 2003–December 2012

In total, these providers made 351,576 referrals to ATAPS between 1 July 2003 and 31 December 2012; 277,307 of referred consumers took up the service. Figures 1, 2 show the number of referrals and sessions delivered by initiative in six-month blocks from July 2003 to December 2012. The half-yearly number of referrals increased steadily over time, beginning with 3434 in July–December 2003 and increasing to 31,544 in July–December 2012. In total, 1,432,130 sessions of care were delivered over the full observation period. Again, the number of sessions increased over time, from 10,247 in July–December 2003 to 149,672 in July–December 2012. A temporary drop in the uptake of ATAPS is observed in the 2007 calendar year following the introduction of the Better Access program.

### Did ATAPS promote a team approach to the management of mental disorders?

The ATAPS guidelines encouraged a team approach to the management of mental disorders by virtue of the fact that both a referrer and provider were required as part of the consumer pathway to care. Furthermore, the consumer was required to return to the GP for a review if more than six sessions were required, then again if more than 12 sessions were required, and at the end of treatment. The review was based on written and/or verbal communication between the referring and treating providers.

Data relating to participation of GPs and other referring and treating mental health professionals indicate that ATAPS promoted a team approach to the management of mental disorders. As noted above, the vast majority of the 32,000 referrers were GPs. However, since 2009 the introduction of the Tier 2 initiatives resulted in involvement of various other referrers, such as staff from emergency departments (n = 171) and community mental health services (n = 106) referring to the *Suicide Prevention* initiative and maternal health nurses (n = 89) to the *Perinatal Depression* initiative. This demonstrates an expansion of the types of referring professionals involved in the team approach to management of mental disorders. Overall, ATAPS was delivered by approximately 7300 mental health professionals, increasing from 609 in 2003–2004 to around 3000 in 2012–2013. Like referrers, the total number of mental health professionals delivering services may not be unique and demonstrated an overall pattern of increase, but at a slower rate. This demonstrates an increase in the number of mental health professionals involved in the team approach to the management of mental disorders. While the minimum dataset did not contain data regarding whether consumers attended a review with their GP, it can be inferred that 16 % of all referrals (i.e., those who received more than the initial six sessions) were likely to have attended a review with their GP.

Secondary analysis of qualitative data from our initial four ATAPS evaluation reports, based on local implementation and evaluation reports [30–32] and an evaluation forum [33], illustrate a consistent theme of improved collaboration and relationships (including communication and knowledge transfer) between GPs and mental health professionals experienced by participating professionals. Evidence of a team approach to ATAPS service delivery was also derived from our evaluations of the various Tier 2 initiatives, which highlighted organisational collaboration within and beyond ATAPS organisations [34–36]. Although the team approach seen in ATAPS was maintained irrespective of the various models of service delivery (method of retention of mental health professionals, method of referral and location of mental health professional), collaboration was found to be optimal in circumstances in which the mental health professional was co-located within the GP’s practice. However, the co-location model is noted to reduce consumer (and GP) choice of mental health professional [30].

### Did ATAPS target services to those individuals requiring primary mental health care who are not likely to be able to have their needs met through Medicare-subsidised mental health services?

People who are typically disadvantaged (e.g., those residing in rural and remote or socio-economically disadvantaged locations, Indigenous people and children) or under-utilise mental health care (e.g., males) are least likely to have their needs met through Medicare-subsidised mental health services. Consumer and session characteristics indicated that over time ATAPS increasingly targeted services to people requiring mental health care who were otherwise not likely to be able to have their needs met.

Over the life of ATAPS, 29 % of all consumers were male; this increased from around 25 % in 2003–2004 to 33 % in 2012–2013, which is positive given that traditionally males with mental disorders are less likely than females to seek mental health treatment from any professionals [37]. The fact that proportionally more males accessed ATAPS initiatives that target homeless people and children, respectively, is probably attributable to a higher prevalence of homeless males than females [38] and boys being more likely to experience externalising behavioural problems that may prompt parents to seek professional help for their children than when internalising behavioural problems are present [39].

Overall, the average age of consumers was 37 years, decreasing from 39 years in 2003–2004 to 35 years in 2012–2013, probably attributable to the introduction of the Tier 2 initiative targeting children in July 2010. For example, while 3.5 % of all consumers were aged 11 years or younger, this increased from less than 1 % in 2003–2004 to over 5 % in 2012–2013. Access to ATAPS by children is important given the opportunity for prevention of, and early intervention for, mental disorders, which may worsen with time or if untreated [40].

Overall, 3 % of consumers were Aboriginal, increasing from around 2 % in 2003–2004 to 6 % in 2012–2013. Torres Strait Islanders comprised 0.5 % of ATAPs consumers, increasing from 0.1 % in 2003–2004 to 1.5 % in 2012–2013. This appears to be a reasonable uptake given that Aboriginal and Torres Strait Islander people accounted for 2.5 % of the Australian population in the 2011 census [41]. In the context that there are large discrepancies in the mental health of Indigenous Australians compared with other Australians [42], the increasing proportion of Indigenous people accessing ATAPS within the most recent financial year is promising [43].

At least 36 % of ATAPS consumers had not previously accessed mental health care; data on previous health care was missing for around 27 % of consumers. This provides further evidence that ATAPS improved access to mental health treatment for those in need who might not otherwise access psychological care. The fact that more consumers (40 %) had previously accessed psychiatric care in recent years is not necessarily negative; it may reflect previous receipt of services via ATAPS specifically or may be related to the high uptake of the suicide prevention initiative, for which it would be expected that consumers would previously have accessed mental health services. Previous access to mental health care may also reflect the increased availability of primary mental health services in general in the recent decade, and/or suggest improved mental health literacy and reduced stigma associated with seeking mental health care, in general, among Australians.

At least 60 % of ATAPS consumers were receiving a low income, as determined by their GPs, increasing from around 54 % in 2003–2004 to 71 % in 2012–2013. Importantly, only 14 % of sessions were associated with payment of a fee by the consumer, with $18.15 (SD = $22.30) being the average session cost to consumers over time. In comparison to earlier years, a smaller percentage, 10 % or less, of consumers were charged a co-payment in recent years (July 2010–December 2013).

Not only has ATAPS reached consumers in major cities (49 %), it has also reached people across the geographical spread, with 24 % in inner regional, 14 % in outer regional, 2 % in remote and 0.5 % in very remote, locations.

The introduction of the various Tier 2 initiatives further enhanced access to services by consumers who were either disadvantaged or at risk of suicide. For example, 3.5 % of all consumers received services via the Suicide prevention initiative, with an increasing proportion in each financial year since the introduction of this initiative in 2008–2009 (2 %) to 2012–2013 (10 %). Around 1 % of all consumers accessed services as a result of psychological sequelae of natural disasters and close to a further 1 % accessed services via the Homelessness initiative. Males were most strongly represented in the Homelessness and Child initiatives (56 and 51 %, respectively).

### Did ATAPS offer non-pharmacological approaches to the management of common mental disorders?

ATAPS consumers typically presented with common mental disorders. The majority of ATAPS consumers were diagnosed with depression (54 %) and/or anxiety disorders (41 %); this clinical profile of consumers remained fairly consistent over time and was aligned with the intended clinical profile of ATAPS consumers, namely people with diagnosed high prevalence mental disorders [7].

Session-based data confirm that ATAPS offered evidence-based non-pharmacological approaches to the management of common mental disorders. Cognitive and behavioural interventions were the most common interventions delivered between July 2003 and December 2012, accounting for 47 and 35 % of all interventions, respectively. Interpersonal therapy and psycho-education were also commonly utilised, accounting for approximately one-quarter of interventions. Narrative therapy, family-based interventions and parent training in behaviour management each accounted for 2 % or less of interventions used in financial years since their introduction in 2009–2010, which is not surprising given that these interventions were originally limited to use in specific Tier 2 initiatives, such as the use of narrative therapy for Indigenous people.

As previously noted, the referring medical professionals, but not the ATAPS providers, were qualified to prescribe psychotropic medication. At the time of referral, around 30 % of all ATAPS consumers were being treated with antidepressants, 6 % with benzodiazepines, 3 % with mood stabilisers and 2 % with phenothiazines.

### Did ATAPS produce better outcomes for individuals with common mental disorders through offering evidence-based short-term psychological interventions within a primary care setting?

The delivery of evidence-based interventions to people with common mental disorders has been confirmed above (Question 4). In terms of the duration or quantity of treatment, on average, interventions were delivered to consumers in five sessions. Evidence has shown a small but significant association between number of psychotherapy sessions and effect size for the treatment of depression, with the size of the effect increasing by only 0.01 with each session beyond four to six sessions [44], indicating that many ATAPS consumers received a dose of psychotherapy equivalent to that shown to be effective in trials. Of the referred consumers who took up the service, 45 % received six or more sessions of evidence-based treatment, with 92 % of sessions delivered in sessions of more than 30 min duration, which is considered to be minimally adequate treatment [45]. These findings should be interpreted in the context that it is possible that the number of sessions delivered to each consumer may be underestimated due to the variable ability of ATAPS-administering organisations to track consumers.

Overall, consumer outcome data was available for 13 % (or 35,072 of 277,307) of consumers who took up the service. Available outcome data for the most commonly used outcomes measures suggest that ATAPS produced positive outcomes for consumers with high prevalence disorders. Table 3 shows the mean differences between pre- and post-treatment scores and the effect size of the difference on the K-10, DASS, MSSI and EPNDS respectively, by initiative. The mean differences were based on total scores for the K-10, MSSI and EPNDS and the sub-scales of the DASS. Across all four measures, the mean difference between pre- and post-treatment scores was statistically significant and indicative of clinical improvement. Although the majority of effect sizes were large (d > 0.8), some moderate effect sizes were observed on the DASS (d < 0.8, d > 0.5), and small effects were observed for children as assessed by the K-10 (d < 0.5) [46]. Overall, with the exception of children assessed using the K-10, these effects are clinically significant in that they exceed those expected from spontaneous remission from depression (d = 0.5) [47].

**Table 3:** Pre- and post-treatment outcome scores on K-10, DASS, MSSI and EPNDS for consumers receiving care through ATAPS by initiative, July 2003–December 2012

	n	Pre-treatment		Post-treatment		Pre-post difference		Effect size
		Mean	SD	Mean	SD	Mean	SD	
*K-10*								
Tier 1	16,693	31.0	7.9	23.0	8.4	8.0**	8.4	0.96
Bushfire	171	31.2	7.4	25.5	7.3	5.8**	6.3	0.92
Child	50	26.0	7.4	22.2	8.3	3.7*	8.7	0.43
Homelessness	51	31.2	9.2	26.8	9.4	4.4**	6.5	0.67
Perinatal depression	207	30.1	7.7	20.5	8.0	9.6**	9.5	1.04
Suicide prevention	324	35.3	7.1	25.8	9.1	9.5**	9.7	0.98
*DASS-anxiety*								
Tier 1	11,544	16.3	9.8	10.5	8.9	5.8**	9.0	0.65
Perinatal depression	389	13.4	9.4	7.0	8.0	6.4**	8.8	0.72
Suicide prevention	403	19.9	10.4	11.3	9.5	8.5**	10.8	0.79
*DASS-depression*								
Tier 1	11,640	20.6	11.0	12.5	10.2	8.1**	10.7	0.76
Perinatal depression	400	16.9	10.0	8.2	8.4	8.8**	10.6	0.83
Suicide prevention	412	27.8	10.5	15.2	11.9	12.6**	12.7	0.99
*DASS-stress*								
Tier 1	11,577	22.3	9.9	14.8	9.8	7.5**	10.1	0.74
Perinatal depression	404	20.6	9.5	12.0	9.4	8.6**	10.8	0.79
Suicide prevention	404	26.0	10.2	15.8	11.1	10.2**	12.1	0.84
*MSSI*								
Suicide prevention	375	14.7	10.9	4.6	7.5	10.1**	10.1	1.00
*EPNDS*								
Perinatal depression	503	16.4	5.2	9.4	6.3	7.1**	7.1	0.99

*ATAPS* access to allied psychological services; *K*-*10* Kessler-10; *DASS* depression anxiety and stress scales; *MSSI* modified scale for suicidal ideation; *EPNDS* edinburgh postnatal depression scale

* *p* < 0.01,** *p* < 0.001

### Did ATAPS complement other fee-for-service programs and address service gaps for people in particular geographical areas and population groups?

The findings described above (Question 3) certainly suggest that ATAPS reached people in particular geographical areas (e.g., regional and remote) and particular population groups (e.g., low income, Indigenous, children, disaster-affected), in turn implying that the ATAPS program potentially complemented the suite of public and private mental health services available in Australia. It makes most sense to examine the complementarity of ATAPS with Better Access, which is the other major mental health reform designed to improve access to primary mental health care.

In absolute terms and in the context of its capped funding, ATAPS delivered less than 10 % of the quantity of services delivered via Better Access program [29]. However, the two programs differ in terms of who they reach and how services are delivered.

Compared with 30 % of consumers of the larger-scale Better Access program in the two most socio-economically disadvantaged quintiles of the Australian population [48], twice as many consumers of ATAPS were in receipt of a low income, with the proportion increasing to 71 % in the most recent financial year. The increasing number of ATAPS consumers in receipt of a low income over time reflects the program’s targeting of those unlikely to be able to have had their needs met through Medicare-subsidised services such as Better Access in which a co-payment is usually required. During 2007–2009, more than 40 % of Better Access services involved a consumer co-payment (average $35) [48], compared with the 14 % of ATAPS consumers who made a co-payment (average $18). ATAPS has been accessed by people across rural and urban locations with around half of its consumers residing outside of major cities. Indeed, our previous findings have shown that compared to the greater absolute reach of Better Access due to its uncapped funding, the targeted nature of ATAPS resulted in a proportionally greater reach across geographic spread, both by rurality and socio-economic disadvantage [29]. Specifically, 45 % of ATAPS and 18 % of Better Access services were delivered in rural areas, and although a significant positive association between socioeconomic profile and number of sessions delivered by both programs was observed, the effect was more than doubled for Better Access (r = 0.22, p < 0.001, ATAPS; r = 0.46, p < 0.001, Better Access) [29]. It is therefore appropriate that the vast majority of ATAPS services were delivered free-of-charge to consumers. Furthermore, the proportion of consumers paying a small co-payment appears to have decreased over time (see Question 3 above).

## Discussion

### Summary of findings

ATAPS represented a major mental health reform through which, for the first time, primary mental health services were made available to consumers entirely or predominantly via government funding. We used 9.5 years of evaluation data to inform the question of whether ATAPS achieved its objectives 11.5 years since its inception. Our findings suggest that the ATAPS program largely met its objectives of offering referral pathways for GPs to support their role in primary mental health care; promoting a team approach to the management of mental disorders; delivering services to individuals who are unlikely to be able to have their needs met through Medicare-subsidised mental health services; offering non-pharmacological approaches to the management of common mental disorders; producing better outcomes for people with common mental disorders through offering evidence-based short-term psychological interventions within a primary care setting; and complementing other fee-for-service programs, such as Better Access, by targeting people in particular geographic locations and population groups.

### Limitations

Findings should, however, be interpreted in the context of several limitations. Our operationalisation of indicators was imperfect since we predominantly relied on routinely-collected data. The minimum dataset from which the data for this study were drawn was a live, national database used by hundreds of users across Australia, and as a result, it is likely that the data may have contained unidentified errors. For example, the number of unique consumers and providers may be over-estimated since Medicare Locals (and formerly Divisions) varied in their ability to identify the same consumer (either referred for additional sessions under the same referral or who had received more than one referral) and/or provider. Conversely, the complexities associated with the transition from Divisions to Medicare Locals may have resulted in an underestimate of the uptake associated with the 2011–2012 financial year. Although only findings for selected outcome measures were reported, we have shown elsewhere that across all of the 13 most commonly used standardised outcome measures, the mean difference was statistically significant and indicative of clinical improvement [19]. Because of the real-world nature of the evaluation, it was not feasible or ethical to include a comparison group, which means that improvements cannot definitively be attributed to ATAPS treatment and availability of pre- and post-treatment outcome data was less than optimal. However, where outcome data were available there are indications that ATAPS achieved its overall aim of producing better outcomes in mental health and the effects largely exceeded those expected due to spontaneous remission [47].

### Policy implications

While ATAPS seems to have met its objectives, unlike the much larger Better Access program, its funding was capped, its reach was far smaller, and its cost-effectiveness has been questioned. In terms of the latter, a review of ATAPS indicated that for the 2008–2009 period, the cost of delivery of service to each person, calculated for individual Divisions ranged from $57 to $631 per session [49] compared to an average of around $139 total cost ($118 cost to government) per session with a clinical psychologist through Better Access based on aggregate data in 2009 [48].

The higher cost of ATAPS sessions is attributable to the substantial level of service administration associated with service delivery through primary care organisations. The cost per ATAPS session does not accurately capture the “cost of providing an episode of care in a way which produces effective health outcomes” and variation in cost is also attributed to the “often significantly higher cost of providing services to remote parts of Australia as well as to the additional costs incurred targeting groups of people who are difficult to reach and may not otherwise access mental health services” [49]. The higher cost per ATAPS session afforded it greater flexibility than Better Access in terms of tailoring models of service delivery to suit the local context. For example, organisations administering ATAPS were able to attract mental health professionals to rural and remote locations where there was a shortage of (or no) such providers by offering salaried (rather than contractual) appointments and/or offering (and subsidising travel costs associated with) fly-in-fly-out services.

These differences between the two major Australian primary mental health programs suggest that Better Access, rather than ATAPS (soon to be subsumed by Primary Health Network-commissioned primary care psychological treatment), is likely to be the solution to providing psychological services to the wider Australian population. However, ATAPS (and its future equivalent) has a definite role to play in supporting Better Access and providing a more tailored service for particular at-risk groups.

Access to Allied Psychological Services differed from Better Access in several other important ways. First, despite the Medicare rebate available to consumers of Better Access, mental health professionals are able to charge consumers any amount above the scheduled fee, which may be unaffordable for people on low incomes. The consumer co-payment permissible via ATAPS on the other hand was capped at $30. Second, non-medical professionals were able to refer consumers to ATAPS (but not Better Access), which is particularly advantageous in situations where a consumer does not have a GP either due to their personal situation or location (as may be the case for Indigenous or homeless people, for example). Third, unlike Better Access, when consumers are children, ATAPS offers the option for parents to attend sessions without the child which benefits the child’s outcomes [50]. Fourth, in a calendar year, Better Access consumers are eligible for 10 individual and 10 group sessions while ATAPS offered 6 to 12 individual sessions, with the option of an additional six sessions in exceptional circumstances, and 12 group sessions. The latter was valuable for consumers who required more treatment than that available via Better Access. Finally, treatment via ATAPS was, and Better Access is, delivered face-to-face, but ATAPS additionally provided for options of telephone, videoconference or web-based sessions. The unique service delivery options available via ATAPS, which will continue to be available in its future form, maximise access to mental health treatment for those in need who might not otherwise access psychological care due to unique needs and financial and other barriers.

The future form of ATAPS can maximise opportunities for complementing Better Access by retaining and strengthening its focus on hard-to-reach and disadvantaged populations and via its flexible service delivery options. It will be interesting to see the effects of the reform to flexible funding pools managed by Primary Health Networks for the commissioning of psychological services in primary care settings, which is currently in progress. The objectives of the new Primary Health Networks are to improve the efficiency and effectiveness of health services and improve the coordination of care, and the flexible funding pool is intended to assist these networks to meet local population needs.

## Conclusions

Our findings indicate that the ATAPS program largely achieved its objectives within a decade of operation. The program increasingly delivered services to a substantial number of consumers who were disadvantaged and historically would not have accessed services. Strategies usually involved cognitive and behavioural interventions, typically delivered to individuals in sessions of 1 h in duration. Importantly, the ATAPS program achieved considerable positive clinical outcomes for consumers with recorded outcome data. The socio-demographic data indicate that the Tier 2 initiatives were successful in reaching their intended target consumer groups in that their profiles differed somewhat from Tier 1 consumers. This suggests that Tier 2 ATAPS carved an important niche by successfully addressing unmet needs among hard-to-reach consumers and through means that are not available via the Better Access program. Overall, the ATAPS program has been, and its future form will continue to be, an integral part of the primary mental health care system in Australia. The extent to which the new form of ATAPS complements other fee-for-service programs and addresses service gaps, and its cost-effectiveness in the context of targeting hard-to-reach people and the outcomes achieved, could be further examined.

## Abbreviations

ATAPS: Access to Allied Psychological Services
CBT: cognitive behavioural therapy

## References

[1] Pirkis J, Harris M, Buckingham W, Whiteford H, Townsend-White C (2007). International planning directions for provision of mental health services. Adm Policy Ment Health.

[2] Bower P, Gilbody S (2005). Managing common mental health disorders in primary care: conceptual models and evidence base. BMJ.

[3] Nieuwsma JA, Trivedi RB, McDuffie J, Kronish I, Benjamin D, Williams JW (2012). Brief psychotherapy for depression: a systematic review and meta-analysis. Int J Psychiatry Med.

[4] Moulding R, Blashki G, Gunn J, Mihalopoulos C, Pirkis J, Naccarella L, Joubert L (2007). Optimising the primary mental health care workforce: How effective can psychological treatments for common mental disorders best be delivered in primary care?.

[5] Australian Institute of Health and Welfare (2014). Mental health services in Australia: Background to mental health services in Australia.

[6] Hickie I, Groom G (2002). Primary care-led mental health service reform: an outline of the Better Outcomes in Mental Health Care initiative. Aust Psychiatry.

[7] Australian Government Department of Health and Ageing. Operational guidelines for the Access to Allied Psychological Services initiative. Canberra: Mental Health Services Branch, Mental Health and Drug Treatment Division; 2012.

[8] Andrews G, Henderson S, Hall W (2001). Prevalence, comorbidity, disability and service utilisation: overview of the Australian National Mental Health Survey. Br J Psychiatry.

[9] Meadows G, Singh B, Burgess P, Bobevski I (2002). Psychiatry and the need for mental health care in Australia. Aust N Z J Psychiatry.

[10] Burgess P, Pirkis JE (2009). Service use for mental health problems: findings from the 2007 National Survey of Mental Health and Wellbeing. Aust N Z J Psychiatry.

[11] Meadows G, Liaw T, Burgess P, Bobevski I, Fossey E (2001). Australian general practice and the meeting of needs for mental health care. Soc Psychiatry Psychiatr Epidemiol.

[12] Australian Government (2011). Medicare locals: guidelines for the establishment and initial operation of medicare locals and information for applicants wishing to apply for funding to establish a medicare local.

[13] Horvath J (2014). Review of medicare locals: report to the minister for health and minister for sport.

[14] Commission National Mental Health (2014). The national review of mental health programmes and services summary.

[15] Australian Government Department of Health: Primary Health Networks Grant Programme Guidelines, V1.2. Canberra: Australian Government; 2016.

[16] Littlefield L, Giese J (2008). The genesis, implementation and impact of the better access mental health initiative. Clin Psychologist.

[17] Reifels L, Bassilios B, King K, Fletcher J, Blashki G, Pirkis J (2013). Innovations in primary mental health care. Aust Health Rev.

[18] Hickie I, Pirkis J, Blashki G, Groom G, Davenport T (2004). General practitioners’ response to depression and anxiety in the Australian community: a preliminary analysis. Med J Aust.

[19] Bassilios B, Nicholas A, Reifels L, Machlin A, Ftanou M, King K, Fletcher J, Blashki G, Burgess P, Pirkis J (2013). Evaluating the Access to Allied Psychological Services (ATAPS) program: Consolidated 10-year report.

[20] Department of Health (2010). National male mental health policy supporting document: healthy minds.

[21] Autralian Bureau of Statistics: Australian Standard Geographical Classification (ASGC) Correspondences: Canberra: Australian Bureau of Statistics; 2013 http://www.abs.gov.au/websitedbs/d3310114.nsf/home/correspondences. Accessed 1 May 2013.

[22] Kessler RC, Andrews G, Colpe LJ, Hiripi E, Mroczek DK, Normand S-L, Walters EE, Zaslavsky AM (2002). Short screening scales to monitor population prevalences and trends in non-specific psychological distress. Psychol Med.

[23] Lovibond PF, Lovibond SH (1995). The structure of negative emotional states: comparison of the Depression Anxiety Stress Scales (DASS) with the Beck Depression and Anxiety Inventories. Behav Res Ther.

[24] Miller IW, Norman W, Bishop S, Dow M (1991). The modified scale for suicidal ideation.

[25] Miller IW, Norman WH, Dow MG, Bishop SB (1986). The modified scale for suicidal ideation: reliability and validity. J Consult Clin Psychol.

[26] Cox JL, Holden JM, Sagovsky R (1987). Detection of postnatal depression: development of the 10-item edinburgh postnatal depression scale. Br J Psychiatry.

[27] Antony MM, Bieling PJ, Cox BJ, Enns MW, Swinson RP (1998). Psychometric properties of the 42-item and 21-item versions of the Depression Anxiety Stress Scales (DASS) in clinical groups and a community sample. Psychol Assess.

[28] Brown TA, Korotitsch W, Chorpita BF, Barlow DH (1997). Psychometric properties of the Depression Anxiety Stress Scales (DASS) in clinical samples. Behav Res Ther.

[29] Bassilios B, Pirkis J, Fletcher J, Burgess P, Gurrin L, King K, Kohn F, Blashki G (2010). The complementarity of two major Australian primary mental health care initiatives. Aust N Z J Psychiatry.

[30] Pirkis J, Blashki G, Headey A, Morley B, Kohn F (2003). Evaluating the access to allied health services component of the better outcomes in mental health care initiative: first interim evaluation report.

[31] Morley B, Kohn F, Pirkis J, Blashki G, Burgess P (2004). Evaluating the access to allied health services component of the better outcomes in mental health care initiative: second interim evaluation report.

[32] Kohn F, Morley B, Pirkis J, Blashki G, Burgess P (2005). Evaluating the access to allied health services component of the better outcomes in mental health care initiative: fourth interim evaluation report.

[33] Morley B, Kohn F, Pirkis J, Blashki G, Burgess P (2005). Evaluating the access to allied health services component of the better outcomes in mental health care initiative: third interim evaluation report: benefits and barriers associated with different models of service delivery.

[34] Bassilios B, Pirkis J, King K, Fletcher K, Blashki G, Burgess P (2012). Evaluation of an Australian primary care telephone cognitive behavioural therapy pilot. Aust J Prim Health.

[35] King K, Bassilios B, Reifels L, Fletcher J, Ftanou M, Blashki G, Burgess P, Pirkis J (2013). Suicide prevention: evaluation of a pilot intervention in a primary care context. J Mental Health.

[36] Bassilios B, Reifels L, Pirkis J (2012). Enhanced primary mental health services in response to disaster. Psychiatr Serv.

[37] Parslow RA, Jorm AF (2000). Who uses mental health services in Australia? an anslysis of data from the national survey of mental health and wellbeing. Aust N Z J Psychiatry.

[38] Australian Bureau of Statistics: 2049.0—Census of population and housing: estimating homelessness, 2011. Canberra: Australian Bureau of Statistics; 2011.

[39] Sayal K (2006). Annotation: pathways to care for children with mental health problems. J Child Psychol Psychiatry.

[40] Kessler RC, Angermeyer M, Anthony JC, de Graaf R, Demyttenaere K, Gasquet I, de Girolamo G, Gluzman S, Gureje O, Haro JM (2007). Lifetime prevalence and age-of-onset distributions of mental disorders in the World Health Organization’s World Mental Health Survey Initiative. World Psychiatry.

[41] Australian Bureau of Statistics: 2075.0—Census of Population and Housing—Counts of Aboriginal and Torres Strait Islander Australians, 2011. Canberra: Australian Bureau of Statistics; 2012.

[42] Jorm AF, Bourchier SJ, Cvetkovski S, Stewart G (2012). Mental health of Indigenous Australians: a review of findings from community surveys. Med J Aust.

[43] Reifels L, Bassilios B, Nicholas A, Fletcher J, King K, Ewen S, Pirkis J (2015). Improving access to primary mental healthcare for Indigenous Australians. Aust N Z J Psychiatry.

[44] Cuijpers P, Huibers M, Ebert DD, Koole SL, Andersson G (2013). How much psychotherapy is needed to treat depression: a metareggresion analysis. J Affect Disord.

[45] Harris MG, Hobbs MJ, Burgess PM, Pirkis JE, Diminic S, Siskind DJ, Andrews G, Whiteford H (2015). Frequency and quality of mental health treatment for affective and anxiety disorders among Australian adults. Med J Aust.

[46] Cohen J (1988). Statistical power analysis for the behavioral sciences.

[47] Rutherford BR, Mori S, Sneed JR, Pimontel MA, Roose SP (2012). Contribution of spontaneous improvement to placebo response in depression: a meta-analytic review. J Psychiatr Res.

[48] Harris M, Pirkis J, Burgess P, Olesen S, Bassilios B, Fletcher J, Blashki G, Scott A: Evaluation of the Better Access to Psychiatrists, Psychologists and GPs through the Medicare Benefits Schedule initiative—Component B. An analysis of Medicare Benefits Schedule (MBS) and Pharmaceutical Benefits Scheme (PBS) administrative data. Final Report. Melbourne: Centre for Health Policy, Programs and Economics, The University of Melbourne; 2010.

[49] Australian Government Department of Health and Ageing (2010). Outcomes and proposed next steps: review of the Access to Allied Psychological Services component of the better outcomes in mental health care program.

[50] Bassilios B, Nicholas A, Ftanou M, Fletcher J, Reifels L, King K, Machlin A, Pirkis J. Implementing a primary mental health service for children: Administrator and provider perspectives. J Child Fam Stud. 2016. doi:10.1007/s10826-016-0572-9

